# Supplementary material for: Psychological Impacts of COVID-19 on Healthcare Trainees and Perceptions towards a Digital Wellbeing Support Package
Source: Int J Environ Res Public Health. 2021 Oct 11;18(20):10647. doi: 10.3390/ijerph182010647 (PMC8535361; doi:10.3390/ijerph182010647)
Supplement: Supplementary file 1 [file ijerph-18-10647-s001.zip › revised-Supplementary File S1 Interview topic guide.pdf]

Supplementary file S1: INTERVIEW TOPIC GUIDE

Psychological impacts of COVID-19 on healthcare trainees and perceptions towards a digital wellbeing support package

Questions

1. If we could start with a little background about you – What is your current area of study?
2. Can you tell me a bit about what was it has been like being a healthcare student during Covid-19; what have been the emotional highs and lows of that time?
3. As you will be aware from the media Covid-19 affected some members of the community differently with ethnic minority groups more vulnerable than white groups.
    a. (if ethnic minority group): As a member of an ethnic minority community, were you aware of this at the time and did you have any specific concerns or experiences related to this as a healthcare student?
    b. (if not ethnic minority group): Were you aware of this at the time and did that affect your experiences as a healthcare student during the pandemic in any way?
4. Were you aware of this package before we sent you the link?
5. What are you views on the package overall?
6. Did you learn anything new from this package? If so, what was the most important thing that you learned?
7. Which aspects of the package did you find most useful?
8. Were there any aspects of the package that were less useful?
9. Have you practically used any of the information from this package at work, in your studies or at home?
10. What are your thoughts about your wellbeing and that of other healthcare students in returning to normal studies and placements post-pandemic? How can we best support psychological wellbeing generally?

Close: Is there anything else you would to share with me today?
