# Supplementary material for: Psychological Impacts of COVID-19 on Healthcare Trainees and Perceptions towards a Digital Wellbeing Support Package
Source: Int J Environ Res Public Health. 2021 Oct 11;18(20):10647. doi: 10.3390/ijerph182010647 (PMC8535361; doi:10.3390/ijerph182010647)
Supplement: Supplementary file 1 [file ijerph-18-10647-s001.zip › revised-Supplementary File S2_Survey.pdf]

Supplementary file S2: Survey

Psychological impacts of COVID-19 on healthcare trainees and perceptions towards a digital wellbeing support package

Digital Package Title: Psychological Wellbeing for Healthcare Workers, Mitigating the impact of COVID-19 on Psychological Wellbeing, Version 1.0.

Digital Package Link: https://www.nottingham.ac.uk/toolkits/play_22794

This is an online learning resource designed to support healthcare staff working during and after the COVID-19 pandemic. This resource has been produced in anticipation of the psychological effect of working during this time. This is an open access, free, online resource.
Topics include: Creating a psychologically safe workspace, advice for team leaders, communication and language, accessing support from others, signposting others through psychological first aid, self-care strategies, working in demanding environments, making difficult decisions, and managing emotions such as fear, anxiety, guilt, stress and low mood. It includes signposting to a wide range of resources to support mental health from official sources.

Instructions
Please read the detailed participant information sheet before choosing to take part in this anonymous survey. By completing the survey, you are consenting to take part in this study. The information is being used to gather insight into wellbeing in healthcare trainees, and to evaluate the usage and impact of a digital package to promote psychological wellbeing in healthcare workers during covid-19. Participation is confidential.

PART 1: ABOUT YOU

Q1. What is your age?
16-20 21-30 31-40 41-50 51-65 66+

Q2. What is your gender?
Male Female Non-binary/Gender fluid Prefer not to disclose

Q3. What is your course or training in?
Nursing Midwifery Physiotherapy
Medicine Dentistry Pharmacy
Ambulance
Allied health professions
Other student e.g. PhD (please specify)

Q4. Year of study
1
2
3
4
5
6 or higher

Q5. Have you been working or on placement in a social care setting during the COVID-19 pandemic?
Yes
No

Q5. Have you been working or on placement in the NHS during the COVID-19 pandemic?
Yes
No

Q5a If yes, which area?
Corporate
Estates & Facilities
Clinical Support
Family Health
Cancer and Specialities (CAS)
Medicine
Surgery
Treatment Centre
GP practice
Community
Other (please specify) = Intensive Care Units, Patient Transport Teams, Acute Medical Unit

Q7. Have you worked or been on placement in a COVID-19 high-risk area during the pandemic?

Covid19 Positive Ward
Intensive Care Unit
A&E
Care/residential home
I do not work in a COVID-19 high risk area
Other (specify)

Q10. Ethnicity

White:
White-British
White-Irish
White – any other White background

Mixed
Mixed – White and Black Caribbean
Mixed – White and Black African
Mixed – White and Asian
Mixed – Any other Mixed background

Asian/Asian British
Asian/Asian British – Indian
Asian/Asian British – Pakistani
Asian/Asian British – Bangladeshi
Asian/Asian British – any other Asian background

Black/Black British

Black/Black British – Caribbean
Black/Black British – African
Black/Black British – any other Black background

Chinese and other ethnic background
Chinese
Any other ethnic background

PART 2: YOUR WELLBEING

Q10. In general, how stressful do you find your course/training?
1 = *not at all stressful*, 2 = *mildly stressful*, 3 = *moderately stressful*, 4 = *very stressful*, 5 = *extremely stressful*.

Q11. Taking everything into consideration, how do you feel about your course/training as a whole?
1= extremely dissatisfied, 2 = mildly dissatisfied, 3 = moderately satisfied, 4 = mildly satisfied, 5= extremely satisfied.

Q12. Are you considering leaving your course/training?
1 = yes, 2 = no

Q13. As far as you can recall, has it happened over the previous 12 months that you have gone to work (including placement or studies) despite feeling that you really should have taken sick leave due to your state of health?
1 = no, never, 2= yes, once, 3 = yes, 2 to 5 times, 4 = yes, more than 5 times

Q14. I am enthusiastic about my training
0=Never (never), 1=Almost Never (a few times a year or less), 2= Rarely (once a month or less), 3=Sometimes (a few times a month), 4= Often (once a week), 5=Very Often (a few times a week), 6= Always (every day)

Q15. My training inspires me
0=Never (never), 1=Almost Never (a few times a year or less), 2= Rarely (once a month or less), 3=Sometimes (a few times a month), 4= Often (once a week), 5=Very Often (a few times a week), 6= Always (every day)

Q16. I am proud of the work that I do
0=Never (never), 1=Almost Never (a few times a year or less), 2= Rarely (once a month or less), 3=Sometimes (a few times a month), 4= Often (once a week), 5=Very Often (a few times a week), 6= Always (every day)

About your wellbeing

Q17. I've been feeling optimistic about the future
0= None of the time,1= rarely, 2=some of the time, 3=often, 4=all of the time.
Q18. I've been feeling useful
0= None of the time,1= rarely, 2=some of the time, 3=often, 4=all of the time.
Q19. I've been feeling relaxed
0= None of the time,1= rarely, 2=some of the time, 3=often, 4=all of the time.
Q20. I've been feeling interested in other people
0= None of the time,1= rarely, 2=some of the time, 3=often, 4=all of the time.

Q21. I've had energy to spare
0= None of the time,1= rarely, 2=some of the time, 3=often, 4=all of the time.
Q22. I've been dealing with problems well
0= None of the time,1= rarely, 2=some of the time, 3=often, 4=all of the time.
Q23. I've been thinking clearly
0= None of the time,1= rarely, 2=some of the time, 3=often, 4=all of the time.
Q24. I've been feeling good about myself
0= None of the time,1= rarely, 2=some of the time, 3=often, 4=all of the time.
Q25. I've been feeling close to other people
0= None of the time,1= rarely, 2=some of the time, 3=often, 4=all of the time.
Q26. I've been feeling confident
0= None of the time,1= rarely, 2=some of the time, 3=often, 4=all of the time.
Q27. I've been able to make up my own mind about things
0= None of the time,1= rarely, 2=some of the time, 3=often, 4=all of the time.
Q28. I've been feeling loved
0= None of the time,1= rarely, 2=some of the time, 3=often, 4=all of the time.
Q29. I've been interested in new things
0= None of the time,1= rarely, 2=some of the time, 3=often, 4=all of the time.
Q30. I've been feeling cheerful
0= None of the time,1= rarely, 2=some of the time, 3=often, 4=all of the time.

PART 3: PACKAGE EVALUATION

1) Were you able to access a full functioning package via the link?

Yes/No

2) Which sections did you view? (tick all that apply)

Psychological impacts	_______	Self-care	_______
Psychologically supportive teams	_______	Manage Emotions	_______
Communication	_______	Further resources	_______
Social Support	_______		

3) Did you understand the information provided in this package?

Yes / No

4) Have you gained sufficient knowledge from this resource?

Yes / No

5) Have you practically used any of the information from this package in either your work or home life?

Yes / No

If yes, how? Please comment below:

6) If not yet, do you think this information could have future value?

Yes / No / N/A

7) Could this resource be used by any health care professional?

Yes / No

8) Please rate the level of burden to complete the package (circle response).

1	2	3	4	5	6	7	8	9	10
(1= zero burden									(10 = highest burden)

9) Was engaging with this resource time well spent?

Yes / No

10) Was using this package challenging with regards your own IT skills?

Yes / No

11) This package is free to use – is this acceptable to you?

Acceptable / Not acceptable

12) How do you feel about this resource being available to healthcare workers? Please rate on a scale of 1-10 (circle response).

1	2	3	4	5	6	7	8	9	10
(1= very negative)									(10 = highly positive)

13) Was this package appropriate for your needs?

Yes / No

14) Did this resource contain meaningful information?

Yes / No

15) Was the content of this package useful? Please rate on a scale of 1-10 (circle response)

1 2 3 4 5 6 7 8 9 10

(1= not at all useful) (10 = extremely useful)

16) Was this resource easy to navigate and use? Please rate on a scale of 1-10 (circle response)

1 2 3 4 5 6 7 8 9 10

(1= not at all easy) (10 = extremely easy)

17) Did you experience any technical difficulties in using this package?

Yes / No

18) How relevant is this package to healthcare students?

1 2 3 4 5 6 7 8 9 10

(1= not at all relevant) (10 = extremely relevant)

19) Would you recommend this package to other healthcare students?

Yes / No

20) Is there anything missing that you feel would be important for healthcare students to receive alongside this package to help support their psychological wellbeing?

Please comment below:

Thank you for completing this
