# Supplementary material for: Psychological Impacts of COVID-19 on Healthcare Trainees and Perceptions towards a Digital Wellbeing Support Package
Source: Int J Environ Res Public Health. 2021 Oct 11;18(20):10647. doi: 10.3390/ijerph182010647 (PMC8535361; doi:10.3390/ijerph182010647)
Supplement: Supplementary file 1 [file ijerph-18-10647-s001.zip › revised-Supplementary File S3 Participant ID and details.pdf]

Supplementary file S3: Participant ID and details

	Discipline	Participant ID+
1	Physiotherapy	101FPhysW
2	Nursing	102FNursW
3	Paramedic	103MParaW
4	Nursing	104FNursW
5	Nursing	106MNursM
6	Medicine	107FMedM
7	Other	108FOthM
8	Medicine	109FMedW
9	Medicine	110FMedW
10	Medicine	111FMedW
11	Medicine	112FMedW
12	Medicine	113MMedM
13	Medicine	114FMedM
14	Nursing	115FNursW
15	Other	116FOthW
16	Medicine	117FMedW
17	Medicine	118MMedW
18	Medicine	119FMedM
19	Midwifery	120FMidM
20	Medicine	121FMedM
21	Medicine	122FMedM
22	Medicine	123FMedM
23	Medicine	124MMedW
24	Medicine	125MMedW
25	Medicine	126MMedM
26	Medicine	127FMedM
27	Midwifery	128FMidW
28	Medicine	129MMedM
29	Medicine	130FMedW
30	Medicine	131FMedM
31	Other	132FOthW
32	Nursing	133FNursW
33	Medicine	134FMedM
34	Medicine	135FMedW
35	Medicine	136FMedM
36	Medicine	137FMedM
37	Medicine	138MMedM
38	Nursing	139FNursW
39	Nursing	140FNursW
40	Medicine	141FMedM
41	Medicine	142FMedW
42	Medicine	143FMedW

Note: + Abbreviation used to support quotations. Participant number; F=Female, M=Male. Medicine: Med; Nursing: Nurs; Midwifery: Mid; Physiotherapy: Phys; Paramedic: Para; Other (e.g. health-related PhD): Oth; W; White; M: Minority Ethnic Group.
